# Nest material preferences in wild hazel dormice *Muscardinus avellanarius*: testing predictions from optimal foraging theory

**DOI:** 10.1093/beheco/arad016

**Published:** 2023-03-25

**Authors:** Sarah A Collins, Sarah M Lane, Minako Ishibashi, Tracey Hamston

**Affiliations:** School of Biological and Marine Sciences, University of Plymouth, Plymouth PL4 8AA, UK; School of Biological and Marine Sciences, University of Plymouth, Plymouth PL4 8AA, UK; School of Biological and Marine Sciences, University of Plymouth, Plymouth PL4 8AA, UK; Wild Planet Trust, Paignton Zoo, Totnes Road, Paignton, Devon TQ4 7EU, UK; Wild Planet Trust, Paignton Zoo, Totnes Road, Paignton, Devon TQ4 7EU, UK

**Keywords:** hazel dormouse, *Muscardinus avellanarius*, nesting material, optimal foraging, preference

## Abstract

Obtaining nesting material presents an optimal foraging problem, collection of materials incurs a cost in terms of risk of predation and energy spent and individuals must balance these costs with the benefits of using that material in the nest. The hazel dormouse, *Muscardinus avellanarius* is an endangered British mammal in which both sexes build nests. However, whether material used in their construction follows the predictions of optimal foraging theory is unknown. Here, we analyze the use of nesting materials in forty two breeding nests from six locations in Southwest England. Nests were characterized in terms of which plants were used, the relative amount of each plant, and how far away the nearest source was. We found that dormice exhibit a preference for plants closer to the nest, but that the distance they are prepared to travel depends on the plant species. Dormice traveled further to collect honeysuckle *Lonicera periclymenum*, oak *Quercus robur*, and beech *Fagus sylvatica* than any other plants. Distance did not affect the relative amount used, although the proportion of honeysuckle in nests was highest, and more effort was expended collecting honeysuckle, beech, bramble *Rubus fruticosus* and oak compared to other plants. Our results suggest that not all aspects of optimal foraging theory apply to nest material collection. However, optimal foraging theory is a useful model to examine nest material collection, providing testable predictions. As found previously honeysuckle is important as a nesting material and its presence should be taken account when assessing suitability of sites for dormice.

## Teaser

Hazel dormice prefer to build their breeding nests from particular plants such as honeysuckle bark, beech, and oak. Dormice will travel further and collect more of these plants, even when other nest material is closer to their nest. We assume that there is something special about these plants and they have a higher value to the dormice, and we need to consider these preferences when assessing the suitability of areas for hazel dormouse conservation.

## INTRODUCTION

Selection is expected to drive the evolution of foraging mechanisms that act to maximize fitness, both in terms of energy expended per unit gain, and relative risk of predation, that is, optimal foraging (Emlen 1966; Stephens and Krebs 1986). This can explain selection of food items in terms of which prey to hunt, how far to travel to forage, and when to move on from one patch to the next (MacArthur and Pianka 1966; Pyke et al. 1977). Central Place Foraging Theory (CPFT) makes specific predictions concerning the behavior of animals that return to a central place, like a nest or cache, after each foraging trip, whereby we expect an additional effect of distance from the central place on the size of the load brought back from each trip (Orians and Pearson 1979; Schoener 1979; Kacelnik 1984; Haarberg and Rosell 2006).

Most of optimal foraging theory, including CPFT, and empirical studies testing the theory, have focused on foraging for food, but, foraging for nesting material presents a very similar problem for an animal; how to get the “best” nesting material for the lowest expenditure of energy and minimal risk of predation (Hansell 2000; Mainwaring and Hartley 2013). While, unlike with foraging for food, the issue of energy obtained does not arise as the material foraged is not consumed, there may be differences in the benefits obtained from different nest building materials (Hansell 2000; Petit et al. 2002; Mainwaring et al. 2014; Ruiz-Castellano et al. 2018). Decisions about which nesting material to use are therefore likely to depend on; 1) the availability of materials in the environment (and the distance to travel in order to collect that material), 2) predation risk while collecting, and 3) variation in nest material quality (Petit et al. 2002; Mennerat et al. 2009a; Mainwaring et al. 2014; Bailey et al. 2016). Therefore, as for optimal foraging decisions, the relative costs and benefits will determine individual decisions on which nesting material to collect, and how much to carry back on each trip (MacArthur and Pianka 1966; Metz et al. 2007).

There have been a number of studies, mainly in birds, on what makes a “good” nest (Hansell 2000). Important factors in determining nesting material, placement and overall nest design have been identified as: 1) the effect on thermoregulation of adults and/or offspring (e.g., Hilton et al. 2004; Gedeon et al. 2010; Dawson et al. 2011), 2) protection from predators (e.g., Gregoire et al. 2003), 3) reduction of parasites (e.g., Gwinner et al. 2000; Lafuma et al. 2001; Soler et al. 2010; Scott-Baumann and Morgan 2015), and potentially 4) attractiveness in sexual selection (e.g., Soler et al. 1998; Brouwer and Komdeur 2004). Depending upon which nest function is more important for a species, different materials are likely to have a higher value. Thermal properties of nests are affected by overall design and the presence of feathers (Hilton et al. 2004), while anti-parasite properties are linked to the inclusion of specific plants (Clark and Mason 1985; Gwinner et al. 2000; Mennerat et al. 2009b). Regardless of which functions are important, the problem is the same, the cost of collection of the material must be traded off with the benefit obtained.

There have been very few studies that have assessed exactly how transport costs affect the collection of nest construction material, but those available show an effect of costs on individual decisions. For example, hamsters conduct fewer trips, and transport larger loads per trip when nesting material is further away (Guerra and Ades 2002). Marsh harriers, *Curcus aeriginous*, minimize material collection costs while still ensuring the nest’s structural quality by changing to less preferred materials when preferred material is further away (Staneviclus and Baleviclus 2005). The most comprehensive study of nesting material use to date was conducted on Cape weavers, *Ploceus capensis*, showing that nest construction is a complex task whereby individuals take into account the material’s structural properties, proximity to the nest site, and its value to the nest (Bailey et al. 2016). However, to date there have been no studies that explicitly test the trade-off between travel costs and material collection in the wild, which would allow us to test when less preferred material is substituted for more preferred material across a range of possible nesting materials.

The hazel dormouse is an endangered UK mammal (Bright and Morris 1995) which has been the subject of a number of conservation efforts over the past 40 or so years. Like other small nocturnal mammals, hazel dormice construct nests that serve as refuges from predators and aid in thermoregulation (Hayward and Rosentreter 1994; Ellison 1995; Deacon 2006), with both males and females building (sometimes multiple) nests throughout the year (Juskaitis 2008). Conservation efforts have largely centered on the placement of nest boxes in the hazel dormouse’s optimal habitat, traditionally coppiced ancient woodland where the variety of plant species in the under-storey provides food required by the dormouse during spring and summer, and enough food in autumn to allow hibernation (Bright and Morris 1995; Bright et al. 2006). Dormice build both summer (for shelter and breeding) and winter (for hibernation) nests and there are differences in their construction and placement. Winter nests tend to be composed of fewer different types of material (Gubert et al. 2022), perform a different function, that is, thermoregulation, and are also built close to the ground (Bright and Morris 1995). However, the availability of nest building material at both times of year may be vital to their success.

The plant species that are used as nesting materials (both for summer and winter nests) are often taken from the nearest available vegetation (Zaytseva 2006; Juskaitis 2008; Bracewell and Downs 2017; Gubert et al. 2022). However, there are many cases where the leaves of the tree on which the nest is located are not the dominant material (Juskaitis 2008). Thus, there is a clear indication that hazel dormice are not only willing to travel some distance from the nest box to obtain nest material, particularly for summer nests (Bracewell and Downs 2017), but also that they prefer certain species over others (Wachtendorf 1951; Bright and Morris 1991; Juskaitis 2014; Čanády 2015). A previous study on the winter hibernation nests of hazel dormouse, *Muscardinus avellanarius,* found that within the 3m radius assessed, some materials were collected from further away than others (Gubert et al. 2022) suggesting an interaction between plant type and distance traveled. In Britain, nests have been found that were partly constructed from honeysuckle bark in areas where there were no signs of honeysuckle growing nearby (Bright et al. 2006). A study on captive hazel dormouse showed that there was a significant preference for grass as the main summer nesting material (Hagemans et al. 2007). However, in this study, the dormice were in a cage, as part of the reintroduction program, with nesting material provided on the ground. However, in the wild we would expect little use of grass in summer nests, which tend to be higher above the ground compared to winter nests as dormice are arboreal (Bright et al. 2006), although in winter the use of grass is more likely.

Here, we set out to examine the nesting material preferences of wild dormice when building summer nests in nest boxes provided by the Devon Dormouse Group and whether they conform to predictions of optimal foraging when collecting material for nest construction. To do so, we: 1) determined the effect of distance to the nest on a) the type, and b) the amount of material used; 2) determined whether the effort exerted (distance traveled × proportion in nest) in collecting a material varies between plant species, and 3) assessed nest material preference when there are minimal travel costs, that is, a plant is next to the nest.

If all materials are equally useful, then optimal foraging theory would predict that whether a plant is used, or not, would depend solely on distance (Staneviclus and Baleviclus 2005; Mainwaring and Hartley 2013; Bailey et al. 2016), that is, travel associated costs minimized. However, if the dormice exhibit preferences for specific plants, or certain plants are more “valuable” than others, the relationship between distance and use of the material would be more complex, with some plants being used despite greater distances, as their value offsets the increased travel costs as suggested by Bracewell and Downs (2017). When costs are relatively equal, as for the study on captive dormice (Hagemans et al. 2007), or for the wild winter nest study (Gubert et al. 2022), we may see a different absolute preference, as how much is used should depend only on the preference for that material, with no travel cost trade off required.

## METHODS

### Nests

**Figure 1:**
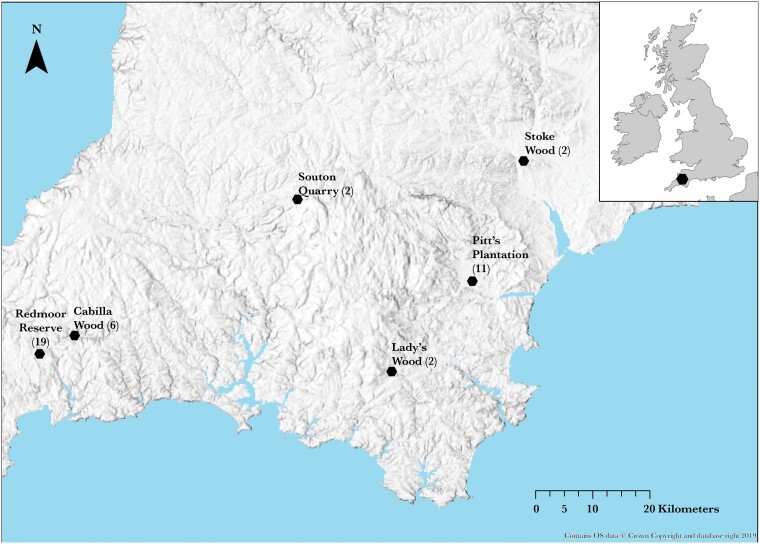
Location of nest sites (number of nests obtained at each site).

Hazel dormice nests were obtained from six different sites (Site) throughout Southwest England (Figure 1) in collaboration with Devon Dormouse Group. A total of 42 summer nests (NestID) were used for data collection and analyses. Nests were located on five different species of tree, hazel, *(Corylus avellana*—30 nests), oak (*Quercus robur*—5 nests), birch (*Betula pendula*—3 nests), beech (*Fagus sylvatica*—2 nests), and hawthorn (*Crataegus laevigata*—2 nests).

All nests were built in wooden nest boxes specially designed for dormice and placed at the recommended height of 1.5–2 m off the ground (Bright et al. 2006), except for one nest from Lady’s Wood that was built in a bird box. Thirty-seven nests were built between April and November 2008 and were entirely taken out from the nest box at the beginning of summer 2009, before the hazel dormice became active and inhabited the nest box. Five of the 11 nests from Pitt’s Plantation were from 2009. For these nests, the nest box it was built in was noted, but since there was a high possibility of it being used by the dormice, due to the time of year, the nests were not taken from the nest box in order to avoid disturbing them, and the data was collected in situ. Composition of nests and proportion of plant materials used at Pitt’s plantation did not differ significantly between years, therefore the data from both years was combined.

### Nest analysis

**Figure 2:**
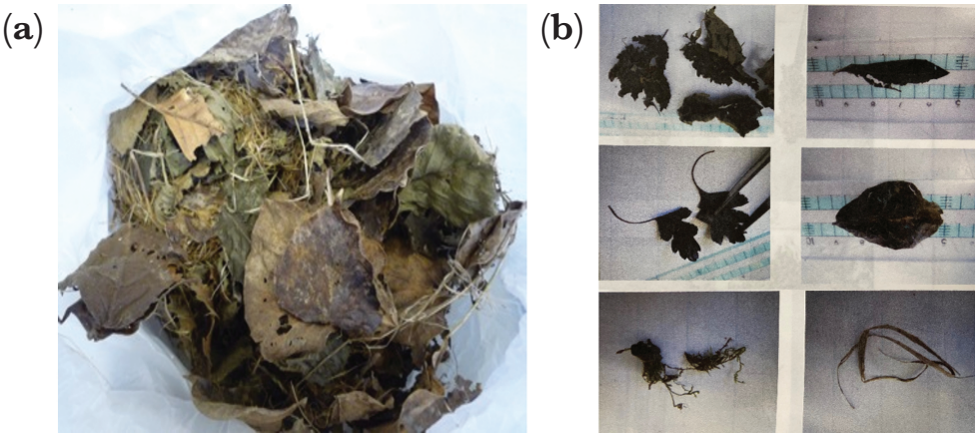
Photos showing a) basic structure of a typical nest, with an outer layer of leaves and an inner woven core of vegetation strands, b) example of nest material extracted from a nest for identification.

Nests were separated by hand, and plant materials (Plant) were identified using a microscope when necessary (Figure 2a and b). For the five nests from Pitt’s plantation that were analyzed at the nest site, we used a twig to move around the nest material to identify the species. Plant materials were separated by species (both bark and leaf), and we recorded, 1) whether a species was used or not (Use), and 2) the percentage of each species in the nest (Perc).

In all nests, hazel bark, honeysuckle (*Lonicera periclymenum*) bark (not leaves), and “grass” (most likely bluebell (*Hyacinthoides non-scripta*), were used as long strips to provide nest structure. The main plants used in lining the nest were oak, birch, bramble (*Rubus fruticosus*), beech, ivy (*Hedera helix*), hazel leaves, and hawthorn. Overall usage of moss was high, but out of 23 nests that contained moss 13 of them had a mass of moss shaped as a cup, which indicates that most likely the dormouse nest was built on top of an old bird’s nest, so moss use was not analyzed further. Other plant species sometimes used in the nests were rhododendron (*Rhododendron ponticum)*, sycamore (*Acer pseudoplatanus)*, blackthorn (*Prunus spinose)*, fern (various species), elder (*Sambucus nigra)*, and mountain ash (*Sorbus aucuparia)*. Each of these was used in only one nest, except for blackthorn which was used in four, so these species were not included in the statistical analysis and the percentage of these materials and moss was combined as “Other.”

### Habitat analysis

We measured the distance (Distance) of the eight most common plant species (honeysuckle, hazel, beech, birch, bramble, hawthorn, ivy, oak) relative to each nest. The distance, measured with field measuring tape, was taken from the circumference of the tree in which the nest box was situated to the circumference of nearest tree of the above eight species, whether or not the species was used in that particular nest. If the tree the nest box was situated on was one of the identified species, the distance for that species was recorded as 0.2 m (assuming that there would be a short travel distance to the leaves); for honeysuckle, the distance to the radius of the tree on which honeysuckle was climbing was measured. Also, since hazel dormice use honeysuckle bark they have stripped off, only mature honeysuckle with a flaky bark surface (i.e., that enabled the dormice to strip the bark) was measured.

Hazel dormice are highly arboreal and move between trees rarely coming down on to the ground (Bright and Morris 1991; Bright et al. 2006), so only those plants that were physically accessible from the tree that nest box was situated in were measured (unlike in Bracewell and Downs 2017 who measured linear distance of a plant to the nest whether or not there was an arboreal route). If a species was absent in the area, that species was recorded as not available. Throughout the analysis hazel leaf and hazel bark are treated as separate data points as they were used for different functions in the nest, filler and structure respectively.

### Statistical analysis

We calculated the percentage of each plant material type in each nest (including all possible plants not just the eight main species). One site did not have any birch or beech, but the rest of the plant species were found at all sites. In total, there were 334 data points from 42 nests.

As hazel and honeysuckle were both used as a core structural material, we conducted a Chi square test (R Studio: Pearson’s) on whether each was “used” versus “not used” across the 42 nests to determine preference for structural material.

#### Plant use and distance

Not all plants were used, even when available in the area (Table 1), so the data contained an extremely high number of 0s for Use in the nest. Therefore, a hurdle analysis was conducted splitting the data into two analyses (Duan et al. 1983; Mullahy 1986; Zuur et al 2009). First, a General linear model (GLM), with a binomial error distribution (using R package *Lme4*; Bates et al. 2015) was used to determine whether material Use (response variable: 0 = no,1 = yes) was affected by 1) the distance to the plant from the nest (“Distance”)—for plants available at that site, 2) plant species (“Plant”); 3) their interaction or 4) site of nest (“Site”), and 5) Nest ID.

**Table 1:** Number of nests (*N* = 42) containing the eight most common plant species, and mean % used in the nests (hazel leaf and bark measured separately)

Plant	Material use (*N* of nests)	Mean % used in nest
Bramble	9	3.15
Beech	23	15.00
Birch	19	6.47
Hazel leaf	28	16.49
Hazel bark	7	4.23
Honeysuckle bark	34	27.48
Hawthorn	7	1.32
Ivy	25	9.39
Oak	26	9.79
Other	8	6.68

Second, a linear model (LM) regression model (using *Lme4*, Bates et al. 2015) was used to determine the effect of distance to the plant from the nest on the log percentage (LogPerc: log transformed for normality) of that plant used in building the nest. Factors included were as above (Plant, Distance, Site, and Nest ID).

#### Plant preference close to nest

We tested whether dormice exhibit a preference for a particular plant species by comparing the use of plants that were close to the nest, that is, transport costs were minimal (as in Gubert et al. 2022). We did this by conducting a second linear model (*lm*) that included usage data only on plants that were less than 2.6 m from the nest (includes the tree nest is sited in, plus directly adjacent trees only). The percentage of each of these plants used in the nest, LogPerc, was included as the response variable (again log transformed but including 0s,), and Plant, Site, and NestID were included as explanatory variables.

#### Relative effort expended on each plant

To test for differences in effort made for each plant, a new variable “Effort” (proportion in nest x distance) was calculated for each material used (a high score indicating higher use at greater distances from the nest). Effort was then included as the response variable in a general linear model fitted with a gamma error distribution and log link with Plant, Site, and NestID as explanatory variables.

Analyses were run both with and without nests containing more than 30% moss (*N* = 7, nests likely built on top of old bird nests), however as excluding those nests did not affect the outcome of the analysis, we have reported only the results including all nests. For all analyses, NestID was not included as a random factor as this led to a singular fit, with a random effects variance of 0, likely because each plant occurs only once per NestID. Therefore, NestID was included as a fixed factor in all models.

For all models, significance was tested using the *drop1* function in R with a chi-square or *F* test (for GLMs and LMs, respectively). Post-hoc pairwise comparisons of significant factors were conducted using Tukey tests with the R package *emmeans* (Lenth 2022).

All statistical analyses were run in R v3.5.1 (R Core Team 2022, version 4.1.0) and figures were produced using R package *ggplot2* (Wickham 2016).

### Ethics

All nests from 2008 were taken from the nest boxes by the dormice license holders who conduct the checks for each site. Even though none of the hazel dormice or the nest boxes were disturbed during the data collection, nest sites were always visited with a dormice license holder when they conducted their routine monthly check. The on-site analysis of the five nests from Pitt’s plantation was also conducted under the supervision of dormice license holder, with extra attention so as not to disturb any local hazel dormice.

## RESULTS

### Description of nesting materials

**Figure 3:**
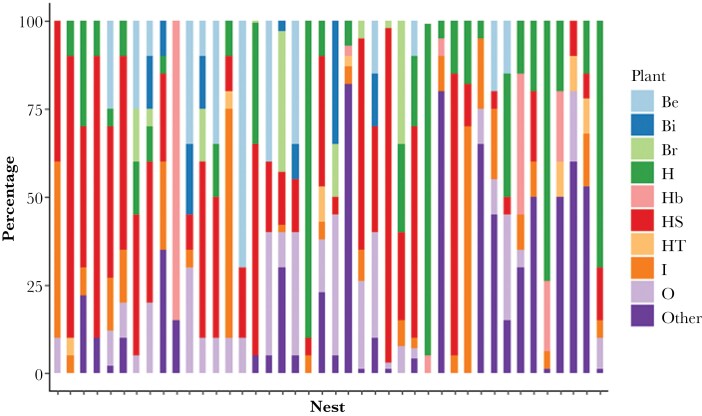
The variation in proportion of plant species used in nests (strands and leaves), including the main plants and “other” for plants used infrequently. High % of “other” typically indicates high use of moss. All nests in Souton Quarry Nature Reserve included more than 30% moss in their construction. However excluding those nests did not affect the outcome of the analyses and there was no effect of site for material use. Be = beech, Bi = Birch, Br = Bramble, H = Hazel leaf, Hb = Hazel bark, HS = honeysuckle bark, HT = Hawthorn, I = Ivy, O = Oak.

Most of the nests were composed of two layers of noticeably different materials; 1) leaves and 2) flexible, narrow, and elongated vegetation strands such as bark and grass. Leaves were used as an outer layer of the nests, and strands were woven as the core part of the nest. Only one nest had no signs of leaves, and one nest was made entirely out of leaves and had no strands. Composition was very variable between nests (Figure 3), but all included at least one of the eight most common species.

Thirty-five nests contained honeysuckle bark as an inner core, seven contained hazel bark, one contained only grass, three contained hazel and grass, one contained honeysuckle and grass, none contained both hazel and honeysuckle. There was a significant preference for honeysuckle bark compared to hazel bark as a core structural material (honeysuckle and hazel were available to all nests: Chi-square test; *Χ*^2^_2_ = 32.21, *P* < 0.001).

### Presence/absence in nest and distance

**Figure 4:**
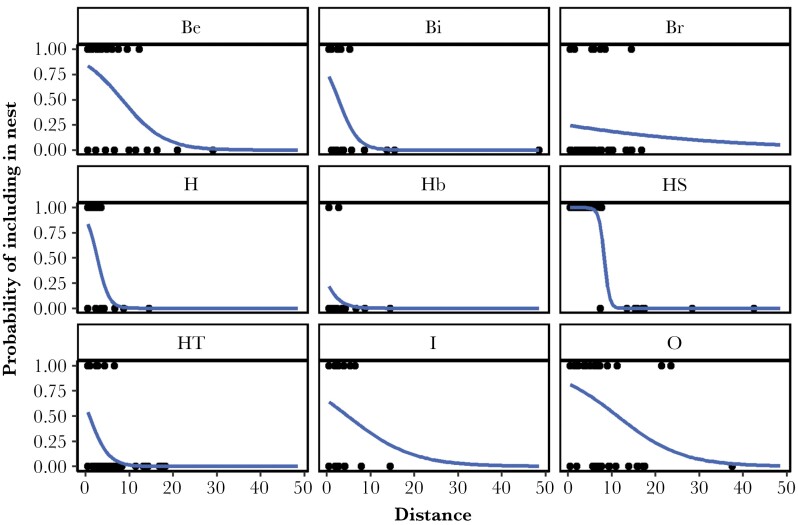
Interaction between the distance to a plant and plant species on the probability of being included in nests. Dots represent raw data, lines show predicted probability of including a plant in the nest based on the general linear model.

There were main effects of Plant, Distance, and Plant × Distance on whether a plant was used or not (glm: plant: LRT = 66.8, *P* < 0.001; distance: LRT = 5.32, *P* = 0.02, plant × distance: LRT = 25.1, *P* = 0.0015, Figure 4). There was no effect of Site (*P* = 0.71) or NestID (*P* = 0.51). Most species were less likely to be used when further away, but the effect varied across nest materials.

### Percentage used in nest

**Figure 5:**
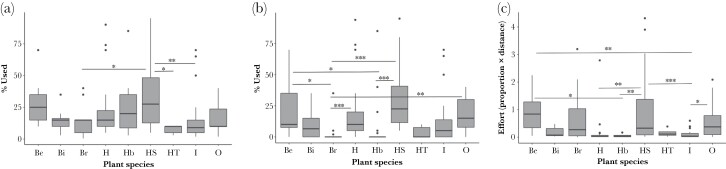
Boxplots of (a) percentage of plant use in nests; (b) percentage used when considering only plants less than 2.6 m from the nest; (c) effort (proportion used × distance) used to collect each species of plant. (*N* = 42). Be = beech, Bi = Birch, Br = Bramble, H = Hazel leaf, Hb = Hazel bark, HS = honeysuckle bark, HT = Hawthorn, I = Ivy, O = Oak.

Plant species had a significant effect on the percentage of plant in the nest (LogPerc) (*F*_8,138_ = 2.32, *P* = 0.03, Figure 5a), but there was no significant effect of Distance or Site (*P* = 0.25, and 0.73 respectively). Honeysuckle use was significantly higher than bramble, hawthorn and ivy (Tukey’s *P* < 0.05).

When considering only plants less than 2.6 m from the nest, there was a significant preference for some Plant species (lm: *F*_8,163_ = 9.7, *P* < 0.0001, Figure 5b). Tukey contrasts showed that honeysuckle, oak, and beech were used at a higher percentage, and bramble and hazel bark were used much less (*P* < 0.001 to *P* = 0.02).

There were significant differences in the amount of effort exerted in order to collect different species of Plant for nest material, (plant: *F*_8,146_ = 4.69, *P* < 0.0001, Figure 5c), there was no effect of Site (*P* = 0.59). Tukey post hoc tests showed in general that more effort was used to collect honeysuckle, beech and oak compared to the other plant species available (*P* < 0.001 to *P* = 0.04).

## DISCUSSION

Our findings show that whether material will be used in a nest, or not, is partially determined by how far away that material is from the nest, consistent with the predictions of optimal foraging theory. However, this effect varies significantly between plant species, with some plants being used even when they are distant from the nest, e.g., oak and honeysuckle. In addition, we found no relationship between the relative amount of material used in the nest and distance to that plant species from the nest, in contrast to what would be expected under optimal foraging theory (specifically, central place foraging models). Furthermore, when looking at the overall effort used to collect different plants, and how much of a plant was used when travel costs were minimum (i.e., considering only plants less than 2.6 m away from the nest), there were also strong preferences for particular species. Honeysuckle bark as a core construction material, and oak and beech as leaf matter were all used in relatively high amounts, even when at greater distances from the nest than other plants. This suggests that nest material collection does not entirely follow predictions from optimal foraging theory due to strong preferences for specific nest materials which likely have more value as a nest material.

The fact that foraging for nest material is only partially consistent with optimal foraging theory is not completely unexpected given there are clear differences between foraging for nest material and foraging for food (Ruiz-Castellano et al. 2018). For example, for some nest materials there may be a minimum amount needed to fulfill the necessary function (Clark and Mason 1985; Lafuma et al. 2001; Soler et al. 2010) unlike foraging for food, when presumably more is always better. However, using optimal foraging theory to make predictions is a useful method for identifying preferences in contexts outside of foraging (Guerra and Ades 2002; Bailey et al. 2016).

Many studies have used a similar approach to ours to investigate habitat preferences (Johnson 1980; Beyer et al. 2010; Lele et al. 2013). Here use of a habitat (nesting material in our study) is considered selective if it is used disproportionately compared with its availability (distance in our study), that is, the amount of that habitat accessible to the animal (Aarts et al. 2008; Beyer et al. 2010). Identifying resources that are used disproportionately relative to their abundance tells us that the resource, for example, plant, is important or has a higher value to the animal (Johnson 1980). As required in order to fully assess preferences (Beyer et al. 2010), we have data on the availability (i.e., distance) of both used and unused material, so that we can assess non-random use of resources (Johnson 1980), that is, preference. The interaction between distance and plant use, and relative use of plants in the nest showed that some plant species are indeed used disproportionately compared to availability. We suggest that this is because those plant materials are more important/useful as a nest material to the dormouse, which would be interesting to research further.

Being able to quantify preference within a habitat, taking into account availability, provides us with valuable insights into the trade-offs the dormice are making when it comes to collecting nest material. The different measures of preference (distance from which a plant is collected, overall effort expended upon collection, and usage when a plant is close) were consistent in terms of which plants were preferred: honeysuckle, oak, and beech. Bracewell and Downs (2017) also found honeysuckle and beech were used in summer nests even when at greater distances than other species. In winter nests, honeysuckle, grasses, ferns, and bracken have been shown to be collected at greater distances from the nest (but all measures were within a 3 m radius of the nest), and no use of material outside the 3 m zone was found (Gubert et al. 2022). Interestingly, hazel leaf is not commonly used despite the species name.

In our study, all but two plants were available in each location and the nest construction components did not differ by site, suggesting that nesting material preference is consistent across the Devon populations we studied. In other locations across Europe, different plants have been found to be commonly used in dormouse summer nests (see references in Juskaitis 2008), for example, bramble and European hornbeam *Carpinus betulus* (Čanády 2015), beech (Wachtendorf 1951, Möckel 1988, Vilhelmsen 1996), oak (Airapetyants et al. 1983), but these studies did not assess availability of alternative materials within the habitat. Variation in nest construction material used across habitats is likely due to different plant species being available, and a number of broad-leaved trees may be equally suitable in place of the preferred oak and beech that we found. However, without assessing whether use of plant material is non-random in relation to distribution of material we cannot determine preference, only usage (Johnson 1980). Our study allows us to determine non-random use of material in summer nests, and therefore explicitly assess preferences.

Although dormice clearly prefer some plant species over others, we are unable to determine the motivation, or behavioral mechanism underlying those preferences. Determining the reasons why a certain construction material is preferred is beyond the scope of the current study and thus our conclusions must remain somewhat speculative. Usually, core structure and filling are needed to fulfill nest functions (Juskaitis 2008, Čanády 2015), and clearly honeysuckle is preferred for structure (Bright et al. 2006; Bracewell and Downs 2017; Gubert et al. 2022). Hazel bark is much narrower and shorter, once processed by the dormouse, and is also less flexible compared to honeysuckle bark (Hamston, pers comm) and thus it may require more time and effort to gather the same amount of hazel. Alternatively, honeysuckle bark may provide a better core structure due to its length and how easy it is to weave. In many studies, including in captivity (Hagemans et al. 2007), grasses are also used as structural material (Wachtendorf 1951; Vilhelmsen 1996; Eden & Eden 2003). Whether the preferred materials perform other functions such as being anti-parasitic (e.g., Clark and Mason 1985; Gwinner et al. 2000; Mennerat et al. 2009b) would be useful to research further through detailed analysis of the properties of the plants used in the nests.

In terms of leaves, there are several possible reasons for why dormice might exhibit a preference for some plant species over others, as mentioned above, some leaves may contain higher levels of anti-parasitic compounds (Clark and Mason 1985; Lafuma et al. 2001) that provide protection or provide better thermoregulation of the nest (Ellison 1995; Mertens 1997). However, further work is required to quantify specific differences in plant compounds in the local habitat and to determine thermal differences in dry leaves. Dormice collect the leaves when green (Juskaitis 2008), but it is assumed leaf thermal properties improve as they dry out. It would be interesting to see what criteria dormice use when collecting leaves and whether these criteria provide the dormice with information on their thermal potential.

Although our results demonstrate a clear preference for certain nesting materials, we were unable to control for the availability of the specific mix of accessible plants, and there could be interactions between plant material distances, for example, perhaps beech is used more often when oak is further away, as both are expected to perform similar functions. The interrelationships between the distances of all the different plants available would be very complex to model. Five of the 42 nests contained a lot of moss, likely due to the fact they were constructed above an old bird’s nest, but whether the presence of pre-existing material has any impact on subsequent nest construction is unclear. Finally, for logistical reasons, that is, being able to find nests, we investigated nests constructed in nest boxes, which could differ somewhat in construction material used compared to “natural” nests which have no external walls.

Overall, our study confirms that when assessing suitability for dormice in UK woodland, all plants are not equal. Honeysuckle is a very important component of nests, as are beech and oak. Although hazel trees may be preferred for the site of the nest, it does not appear to be used very often for construction. In addition, we have shown that looking at use in relation to availability of material for nest construction allows us to determine importance and preference more clearly than looking at overall use alone.

## Notes

Many thanks to Stephen Caroll, Janet Chapman, Jackie Gage, Cheryl Mills, Chris Moulton, Sue Smallshire, and Jenny Stuart for all the nests they contributed to this project, as well as for their help out in the field, especially to allow measurement during their dormouse surveys. Also thank you to Dr. Stephen Burchett for help in identifying the plants, Dr. Rob Puschendorf for producing the map, and to Prof. Mark Briffa for help with the data analysis.
